# Female cricket pace bowling: kinematic and anthropometric relationships with ball release speed

**DOI:** 10.17159/2078-516X/2023/v35i1a15080

**Published:** 2023-06-05

**Authors:** C Lyons, PJ Felton, C McCabe

**Affiliations:** 1 School of Sport, Ulster University, Belfast, Northern Ireland; 2 School of Science & Technology, Nottingham Trent University, England

**Keywords:** fast bowling, technique, size, shoulder, height

## Abstract

**Background:**

Despite an increase in the professionalism and participation of female cricket, the coaching of female pace bowling is still reliant on male-derived knowledge.

**Objectives:**

To investigate the association between key male-derived kinematic and anthropometric parameters and ball release speed (BRS) in female pace bowlers.

**Methods:**

Eleven female pace bowlers participated in this study. BRS, and four anthropometric and five kinematic parameters were determined. Stepwise linear regression and Pearson Product Moment correlations were used to identify anthropometric and kinematic parameters linked to BRS.

**Results:**

The best predictor of BRS explaining 89% of the observed variance was the bowling shoulder angle at ball release. The best anthropometric predictor of BRS was height explaining 53% of the observed variance. Other parameters correlated with BRS included: run-up speed (r = 0.75, p = 0.013) and arm length (r = 0.61, p = 0.046). When height was controlled for, the front knee angle at front foot contact was also correlated to BRS (r = 0.68, p = 0.044). No relationship was found between trunk flexion and BRS.

**Conclusion:**

Faster BRS were characterised by faster run-up speeds, straighter front knees, and delayed arm circumduction similar to male pace bowlers. The lack of relationship between trunk flexion and BRS may highlight female pace bowlers adopting a bowling technique where BRS is contributed to by trunk rotation as well as trunk flexion. This knowledge is likely to be useful in the talent identification and coaching of female pace bowlers.

Cricket is a sport played between two teams where players assume batting, bowling, or fielding roles which dictate their main responsibilities within a game. A bowler’s primary objective is to dismiss the batters on the opposing team and restrict the number of runs they score. Pace bowlers aim to dismiss batters by maximising their ball release speed (BRS) and decreasing the time they have to react to the delivery of the ball and execute the correct shot.^[1]^ Research in pace bowling has therefore focused on investigating variables associated with BRS to inform coaching practice.^[1–11]^

Previous studies have investigated the impact on BRS of kinematic variables including the run-up,^[1–4]^ the front leg kinematics,^[1,3,5–7]^ the motion of the thorax,^[1,3,6,7]^ and the position of the bowling arm.^[1,3,7]^ The bowlers with the fastest BRS have exhibited faster run-up speeds, as well as straighter front knee kinematics, more trunk flexion and delayed arm circumduction from front foot contact to ball release. The effect of anthropometric parameters on BRS have also been investigated with variables including limb length, body composition, height, and mass.^[2,8–11]^ Positive associations have been reported between limb length and height with longer levers associated with increased angular velocities and subsequent BRS.^[2,8–11]^ Despite previous research investigating the relationships between kinematic and anthropometric variables with BRS, the findings are almost exclusively derived from male populations.

Although female participation and professionalism has increased in recent years, there remains a lack of research into female cricket.^[12]^ Early research comparing male and female pace bowling biomechanics has found differences in ball release speed, run-up speed, and the kinematics at back foot contact, front foot contact and ball release.^[13]^ These findings suggest females may utilise a different movement pattern to generate BRS compared to males, and that extrapolating information from research conducted on male pace bowlers to coach female pace bowlers is potentially erroneous.^[14]^ Nevertheless, coaches working with female pace bowlers are restricted due to the current coaching pedagogy being derived on studies conducted using male bowlers. This study therefore aims to investigate whether previously reported kinematic and anthropometric relationships with BRS in male pace bowlers exist for female pace bowlers.

## Methods

### Participants

Eleven high performance female pace bowlers participated in this study. All bowlers were right-handed and senior members of either Irish International or Interprovincial team members. The testing procedures (as approved by the Ulster University Research Ethics Committee) were explained to all bowlers, health history screening questionnaires were completed, and informed consent was obtained. Prior to data collection, all bowlers were deemed fit to bowl and a thorough warm-up was conducted allowing participants to familiarise themselves with the testing environment.

### Data collection

Kinematic data were collected at an indoor facility where bowlers were able to use a full length run-up to deliver a 141,75 g female cricket ball on a standard sized cricket pitch. Sixteen tape markers (20 × 20 mm) were placed bilaterally on the acromion, olecranon, mid-point of the thoracic cage (approximately T8), anterior superior iliac spine, lateral malleolus, medial malleolus, the carpus, and the armpit of each participant. Six maximal effort deliveries targeted at a good length were recorded using two iPhone 11 video recorders (Apple Inc, California, USA) capturing at 240 Hz. Each iPhone was mounted on a tripod at a height of 0.91 m and placed 6 m perpendicular to the sagittal plane on either side of the bowling crease, so the optical axis aligned with the plane of motion of the bowler during the delivery.^[5]^ A batter was not involved to prevent distraction from bowling with maximal effort,^[4]^ but a target was placed on a good length.^[2]^ Between deliveries bowlers were instructed to rest until they felt ready to bowl another delivery at maximal effort.^[8]^ BRS was measured using a radar gun (Bushnell, 10-1911, USA) positioned 2 m behind the stumps at the bowler’s end. Run-up speed was determined using two pairs of photocell timing gates (TCi system, Brower Timing Systems, Utah, USA). Each pair of timing gates was set to hip height (approximately 1 m) and placed 5 m apart (0.5 and 5.5 metres behind the bowling crease).

Four anthropometric measurements were also taken. Height (m) and weight (kg) were recorded using a stadiometer and digital scales respectively, as well as front leg and bowling arm length (m). Leg length was measured from the anterior superior iliac spine to the lateral malleolus, and arm length from the acromion process to the tip of the digitus medius.

### Data analysis

The variation in BRS and each of the four kinematic parameters (run-up speed, front knee angle at FFC, shoulder angle at BR, and trunk flexion from FFC to BR) was assessed using analysis of variance (ANOVA). The between-trial variability was compared with the between-bowler variability and was found to be much smaller with interclass correlation coefficients (ICC) ranging from 0.81 to 1.00 (mean: 0.92). The three deliveries were consequently averaged for each kinematic parameter to provide representative data for each bowler and put forward for statistical analysis.^[1]^

### Statistical analysis

All statistical analyses were performed within SPSS v.28 (SPSS corporation, USA). An alpha value of 0.05 was used as a threshold for significance with no adjustment made for multiple comparisons due to the investigatory nature of the study.^[15]^ Forward stepwise linear regressions were used to identify the anthropometric and kinematic (independent) variables which best explained the variation in BRS (dependent variable). Pearson’s product moment correlation was used to identify ‘candidate’ variables for input into the regression models with an alpha value below 0.05 required for selection. To ensure all potential candidate variables were identified, kinematic variables were also eligible for selection if Pearson’s product moment correlation alpha values were below 0.05 when anthropometric candidate variables were used as a covariate. Predictor variables included in the two individual regression models (anthropometric and kinematic) were put forward as candidate variables to an overall regression model. Entry requirements for the inclusion of a parameter into the regression equation was p < 0.05, with a removal coefficient of p > 0.10. The regression model was rejected if the coefficient 95% confident intervals included zero, the residuals of the predictor were heteroscedastic or if the bivariate correlations, tolerance statistics or variance inflation factors showed any evidence of multicollinearity.^[16]^ The normality of the standardised residuals was also confirmed by the Shapiro-Wilk test. The percentage variance of the dependent variables (BRS) explained by the independent (kinematic) variables in each regression equation was determined by Wherry’s R^2^ value.^[17]^ This represents an attempt to estimate the proportion of the variance that would be explained by the model had it been derived from the population (elite female fast bowlers) from which the sample was taken. To overcome the potential limitations of stepwise regressions relying on a single best fit model, all possible regression models with the same number of predictor variables were checked.

## Results

The 11 bowlers (age: 22.3 ± 4.7 years; height: 1.68 ± 0.08 m; mass: 73.0 ± 8.3 kg) produced mean BRS of 23.0 ± 1.8 m·s^−1^ in the range: 20.3 – 26.4 m·s^−1^. Descriptive data are reported in Table 1. Two anthropometric (height and arm length) and two kinematic parameters (run-up velocity and shoulder angle at BR) were found to be linearly correlated with BRS (Table 2) and used as candidate variables for the linear regression models. A further kinematic parameter (knee angle at BR) was added as a candidate variable after a linear correlation was observed for BRS when anthropometric candidate variables were controlled for (Table 2).

The candidate variables were investigated for multicollinearity using bivariate correlations. Since arm length was observed to be significantly correlated with height with a Pearson’s correlation coefficient greater than 0.80 it was removed as a candidate variable.^[16]^ All other significant correlations between candidate variables were below 0.80 and were deemed suitable for entry in the forward stepwise linear regression models.

**Fig. 1:**
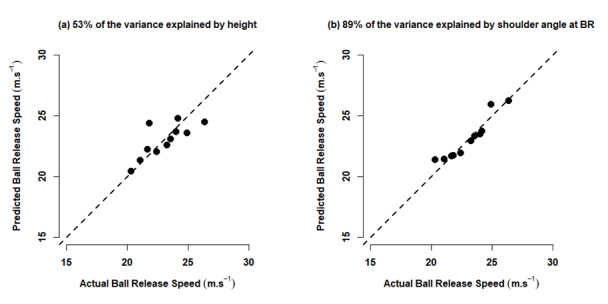
Predicted ball release speed against actual ball release speed for: (a) the anthropometric regression model and (b) the kinematic regression model. With a higher percentage of the variation in ball release speed explained the closer the data points lie to the dashed line y = x (predicted ball release speed = actual ball release speed).

The best anthropometric predictor of BRS was height explaining 53% of the variation in BRS (Table 3, Figure 1a). Greater height characterised the bowlers with the faster BRS. No other candidate variables qualified for entry into the anthropometric regression equation. The best kinematic predictor of BRS was the shoulder angle at BR explaining 89% of the variation (Table 3, Figure 1b). A more delayed bowling arm (greater shoulder angle) at BR characterised the bowlers with the highest BRS. The best overall model predicting BRS mirrored the kinematic model with only shoulder angle at BR entered. A two-parameter model was found when both height and shoulder angle at BR were included in the regression equation (Table 3); however, this was rejected due to the height coefficient 95% confidence interval including zero.

## Discussion

The study aimed to investigate whether previously reported kinematic and anthropometric relationships with BRS in male pace bowlers exist for female pace bowlers. The most important parameter with respect to increasing BRS was the bowling shoulder angle at BR. The fastest bowlers had their arm further back relative to their upper trunk at BR. This aligns with male research which also identifies the shoulder angle at BR to be the best predictor of BRS.^[1]^ A more delayed bowling arm has been proposed to allow greater amounts of trunk flexion, while still allowing the arm to deliver the ball towards the intended target.^[7]^ Consequently, increased trunk flexion has also been linked to greater BRS in male pace bowlers.^[1,3,7]^ No relationship, however, was observed between BRS and trunk flexion from FFC to BR in this study. Female pace bowlers have previously been reported to have longer trunks than their male counterparts.^[11]^ In theory, longer trunks have larger transverse moments of inertia increasing the resistance of the trunk to flex or extend about the pelvis. Previous research has suggested that female bowlers generate BRS using a more rotational technique than males,^[13]^ where a combination of trunk flexion and rotation contribute to the delay in the bowling arm. Further research is required therefore to understand the role of trunk flexion and rotation throughout the bowling action and how this affects delayed arm circumduction in female pace bowlers.

The best anthropometric predictor of BRS was height. The tallest bowlers in this study benefited from an anthropometric effect which aided their generation of BRS. A taller stature has previously been identified as a benefit to male BRS since the longer and heavier segments help increase angular velocity and BRS.^[2]^ The positive correlation between arm length and BRS found in this study and reported in previous male pace bowling research^[2,10]^ also aligns with this theoretical explanation. Nevertheless, care should be exerted when applying this knowledge. Theoretically, taller players need to generate more power to move their longer and heavier segments at similar speeds to their shorter counterparts. This relationship is not linear and therefore optima exist where the increase in size cannot be overcome by an increase in muscle power, and BRS is negatively impacted. In other words, bigger is not continuously better, and there is a tipping point based on the ability to maintain the power to weight ratio.

Contrary to previous male derived findings,^[2]^ no significant association was identified between leg length and BRS for the female pace bowlers in this study. Differences in body composition between males and females may explain this. Female pace bowlers have been reported to have shorter legs compared to male pace bowlers.^[11]^ The role of the lower half of the body in generating BRS in pace bowling is to brake the pelvis, converting the linear momentum developed in the run-up into angular momentum about the centre of mass.^[1]^ The optimal technique for converting linear momentum to angular momentum is to adopt a straight front leg.^[1,7]^ In this study, greater run-up speeds and straighter front knee angles at FFC (when height was controlled for) were associated with increased BRS. These findings are similar to those previously observed in male pace bowlers.^[1–7]^ The mean run-up speed (5.05 m·s^−1^) is slightly slower than those previously observed (females: 5.31 m·s^−1^; males: 5.76 m·s^−1^) ^[13]^;however, this difference is likely to be a consequence of adopting a different measurement approach and limits comparison. Increased run-up speeds were also associated with taller bowlers and the association between run-up speed and BRS was weakened when height was controlled for. This indicates that individual-specific optimal run-up speeds exist and are most likely based on height. Future investigations exploring BRS may need to control for height (or a similar anthropometric parameter) if the participants' anthropometric parameters are not homogeneous.

No relationship between BRS and body mass was observed in this study. Although using body mass can be misleading, due to large variations in underlying variables,^[18]^ no relationships between BRS and the measurements of fat mass, bone mass, muscle mass, and body composition have been observed in male pace bowlers.^[9]^ While the findings of this study suggest the relationship between BRS and body mass is similar for males and females, it has been proposed that differences in body composition may cause the gender gap in BRS.^[12]^ While understanding this was beyond the scope of this study, it is recommended that future studies investigate more detailed anthropometric measures and their effect on technique as well as BRS.

This study has some limitations. Firstly, the sample size of eleven female pace bowlers (while still relatively large for this population) limits the power of the statistical tests conducted. Secondly, the two dimensional analysis employed due to the testing environment are secondary in terms of accuracy to more complex three dimensional approaches available in the laboratory. Finally, the aim of this investigation was to determine whether relationships between kinematic variables previously linked to BRS in male fast bowlers exist in female pace bowlers. A greater number of kinematic parameters are required to fully appreciate the characteristics of technique which influence BRS in female pace bowling. While the findings of this study indicate some alignment between male and female pace bowling techniques to maximise BRS, this may occur due to female pace bowlers being coached based on male pace bowling philosophy, and not because this is the optimal method to generate BRS for female pace bowlers. Further research is required to understand the optimal technique to maximise BRS in female pace bowlers, ideally using three dimensional motion capture and sample size with large statistical power.

## Conclusion

In conclusion, this study investigated whether kinematic and anthropometric relationships with BRS in female pace bowlers are similar to those previously reported in male pace bowlers. The findings highlight that greater BRS were characterised by increased height and larger bowling shoulder angles at BR. In addition, evidence highlighted relationships between greater BRS and straighter front knee angles at FFC and run-up speed.

While these relationships are similar to those observed in male pace bowlers, the lack of a relationship between trunk flexion and BRS in this study may add evidence to the theory that trunk rotation is important in developing BRS in female pace bowlers. This study also highlighted the relationship between height and run-up speed, as well as BRS, indicating that each individual will have an optimal run-up speed, and a potential BRS ceiling based on their height. These findings are likely to be extremely useful in the development of knowledge regarding female pace bowling, especially coaching and talent identification. Future research should aim to build on these findings adopting more complex methodologies and larger sample sizes to improve statistical power in order to develop cause and effect relationships for the female pace bowling action. In particular, understanding the role of trunk rotation and flexion of bowling shoulder arm delay and the generation of BRS.

## Figures and Tables

**Table 1:** Kinematic and anthropometric parameters: range, mean, standard deviation (n=11)

Parameter	Mean ± SD	Range
**Anthropometric**		
Leg length (m)	0.90 ± 0.08	0.73 – 1.00
Arm length (m)	0.71 ± 0.05	0.64 – 0.82
**Kinematic**		
Run-up velocity (m·s^−1^)	5.05 ± 0.61	3.72 – 5.77
Front knee angle at FFC (°)	167.0 ± 2.9	160.7 – 171.4
Bowling shoulder angle at BR (°)	180.3 ± 7.7	172.7 – 194.4
Trunk flexion from FFC to BR (°)	43.5 ± 8.5	24.4 – 57.2

FFC, front foot contact; BR, ball release

**Table 2:** Bivariate and partial correlations between BRS and the anthropometric and kinematic parameters (n=11)

Parameters	r	95% CI		p

		Lower	Upper	
**Bivariate correlations**				
Anthropometric				
Height (m)	0.76	0.29	0.93	0.007
Mass (kg)	0.07	−0.56	0.64	0.85
Leg length (m)	0.24	−0.42	0.73	0.48
Arm length (m)	0.61	0.02	0.89	0.05
Kinematic				
Run-up velocity (m·s^−1^)	0.75	0.22	0.94	0.01
Front knee angle at FFC (°)	0.49	−0.16	0.84	0.13
Bowling shoulder angle at BR (°)	0.95	0.82	0.99	<0.001
Trunk flexion from FFC to BR (°)	−0.19	−0.71	0.46	0.57
**Partial correlations**				
Covariate: height				
Run-up velocity (m·s^−1^)	0.41	−0.58	0.80	0.28
Front knee angle at FFC (°)	0.68	0.18	0.98	0.04
Bowling shoulder angle at BR (°)	0.94	0.77	0.99	<0.001
Trunk flexion from FFC to BR (°)	−0.05	−0.86	0.93	0.90
Covariate: arm length				
Run-up velocity (m·s^−1^)	0.60	−0.42	0.88	0.09
Front knee angle at FFC (°)	0.67	−0.28	0.99	0.05
Bowling shoulder angle at BR (°)	0.94	0.74	0.99	<0.001
Trunk flexion from FFC to BR (°)	−0.07	−0.94	0.77	0.87

FFC, front foot contact; BR, ball release; CI, confidence interval

**Table 3:** Forward stepwise linear regression models predicting ball release speed (n=11)

Model	Candidate variables	Coefficient	95% CI		p	Explained variation (%)

			Lower	Upper		
Anthropometric	Height (m)	17.379	5.429	29.329	0.010	53
	Constant	−6.361	−26.578	13.855	0.489	

Kinematic	Shoulder angle at BR (°)	0.224	0.168	0.279	<0.001	89
	Constant	−17.294	−27.280	−7.308	0.004	

Overall	Height (m)	5.776	−0.068	11.621	0.052	93
	Shoulder angle at BR (°)	0.185	0.125	0.246	<0.001	
	Constant	−20.131	−29.014	−11.247	<0.001	

BR, ball release; CI, confidence interval
